# Making acute ischemic stroke thrombi visible in MRI imaging

**DOI:** 10.1038/s41598-024-62985-1

**Published:** 2024-05-29

**Authors:** Aglaé Velasco Gonzalez, Boris Buerke, Dennis Görlich, Cristina Sauerland, Manfred Fobker, Astrid Jeibmann, Walter Heindel, Andreas Faldum, Werner Paulus, Harald Kugel

**Affiliations:** 1 Clinic for Radiology, University of Muenster, Albert-Schweitzer-Campus 1, Gebäude A1, 48149 Muenster, Germany; 2 Institute of Biostatistics and Clinical Research, University of Muenster, Schmeddingstraße 56, 48149 Muenster, Germany; 3 Center for Laboratory Medicine, University Hospital Muenster, Albert-Schweitzer-Campus 1, Gebäude A1, 48149 Muenster, Germany; 4 Institute of Neuropathology, University Hospital Muenster, Pottkamp 2, 48149 Muenster, Germany; 5 Clinic for Radiology, Neuroradiology, University of Muenster, Albert-Schweitzer-Campus 1, Gebäude A1, 48149 Muenster, Germany; 6 Institute of Neuropathology, University of Muenster, Domagkstraße 19, 48149 Muenster, Germany; 7 Clinic for Radiology, Medical Physics, University of Muenster, Albert-Schweitzer-Campus 1, Gebäude A1, 48149 Muenster, Germany; 8 Clinic for Radiology, Neuroradiology, University Hospital of Muenster, Albert-Schweitzer-Campus 1, Building A1, 48149 Muenster, Germany

**Keywords:** Translational research, Experimental models of disease, Stroke, Diagnostic markers

## Abstract

Knowledge of thrombus behavior and visualization on MRI in acute ischemic stroke is less than optimal. However, MRI sequences could be enhanced based on the typical T1 and T2 relaxation times of the target tissues, which mainly determine their signal intensities on imaging. We studied the relaxation times of a broad spectrum of clot analogs along with their image characteristics of three sequences analyzed: a T1-weighted turbo inversion-recovery sequence (T1w Turbo IR), a T1-weighted turbo spin echo with fat suppression (T1w TSE SPIR), and a T2-weighted 3D TSE with magnetization refocusing to remove T1 dependence (T2w TSE DRIVE). We compared their imaging behavior with the intensity values of normal brain tissue using the same imaging protocols as for clots. Each histological and biochemical clot component contributed to each of the relaxation times. Overall, histological composition correlated strongly with T1 times, and iron content, specifically, with T2 relaxation time. Using decision trees, fibrin content was selected as the primary biomarker for T1 relaxation times, inducing an increase. Up to four clot subgroups could be defined based on its distinctive T1 relaxation time. Clot signal intensity in the T1 and T2-weighted images varied significantly according to T1 and T2 relaxation times. Moreover, in comparison with normal brain tissue intensity values, T2w DRIVE images depict thrombi according to the principle of the more fibrin, the higher the intensity, and in T1w TSE, the more erythrocytes, the higher the intensity. These findings could facilitate improvements in MRI sequences for clot visualization and indicate that T2w DRIVE and T1w TSE sequences should depict the vast majority of acute ischemic stroke thrombi as more hyperintense than surrounding tissues.

## Introduction

When treating acute ischemic stroke (AIS), the success of fibrinolysis and endovascular clot extraction seems to be heavily dependent on clot histology^1,2^. Moreover, ideally, different extant techniques for intracranial clot removal may be selected for each individual patient according to the type of clot causing the arterial occlusion to improve the likelihood of successful recanalization^3,4^. Thus, it is noteworthy that clot imaging focused on thrombus characterization has not yet been an important goal in neuroimaging research, especially MRI, where thrombi causing acute ischemic stroke are mostly invisible to ordinary stroke MRI sequences. Considering that MRI imaging, with its complex generation of contrast, is in principle more appropriate for displaying and differentiating soft tissue than CT, it would be advantageous to adapt conventional MRI stroke protocols for enhanced visualization of AIS thrombi and at the same time information on clot composition.

Biological tissues have typical T1 and T2 relaxation times^5,6^, which largely determine their signal intensities in MRI sequences. While both relaxation processes occur simultaneously depending on the MR imaging sequence contrast parameters selected, signal intensity may be more sensitive to T1 or to T2 relaxation times, resulting in T1-weighted (T1w) or T2-weighted (T2w) images. The effect of tissue relaxation times on imaging signal intensities is regulated by adjusting pulse sequence parameters such as repetition time (TR) for T1 and echo time (TE) for T2 times. Thus, the shorter the T1 time of a tissue, the higher the relative signal intensity compared to the surrounding tissue that can be obtained after a specific (short) TR, whereas tissues with long T2 will display high intensity relative to tissues with shorter T2, especially at long TE^7^. In this sense, optimization of MRI imaging sequences is supported by ascertaining the typical relaxation times of the target tissues, i.e. thrombi and surrounding tissues^8,9^.

Acute ischemic stroke thrombi are complex tissues. As products of fluid blood, they share the same constituents, but in different proportions, are more viscous, and have a broad spectrum of physicochemical properties resulting from their diverse composition^3^. Thus, it is to be expected that clot relaxation times vary depending on their histological and biochemical composition, leading to different MRI appearances. Progressive increases in thrombus hematocrit decrease T1 and T2 relaxation times, with a larger effect on T2 than on T1 relaxation^10^. Clot retraction is a natural process that shrinks the size of a clot by expelling serum and compacting the red blood cells (RBC) into a fibrin mesh. This process contributes to decrease the T2 relaxation times and apparent diffusion coefficient (ADC) values^10,11^.

As the primary constituent of RBC, the hemoglobin is a significant contributor to reduction of T2 relaxation times, particularly in its deoxyhemoglobin form, but also to some extent in its oxyhemoglobin form. On the one hand as protein that binds water molecules, thereby reducing their mobility, and on the other hand as oxygen and iron carriers, causing stronger local field variations due to its paramagnetic properties^12–14^. Strictly speaking, deoxygenated blood cells are still slightly diamagnetic, but less diamagnetic than oxygenated blood cells. As a result, the susceptibility difference to diamagnetic tissue water increases, i.e. the field variation around red blood cells increases^15^. Similarly, decreased T1 times are driven by presence of deoxyhemoglobin but even more by methemoglobin. The latter is found at negligible concentrations in oxygenated blood and increases progressively in conditions of deoxygenation. Thus, the presence of methemoglobin plays a relevant role in influencing signal intensities in subacute rather than acute hemorrhage and thrombus^16–18^. Besides, there is some evidence that T1 times could be related to fiber structure, orientation, and grade of permeability to water^11,19^.

**Figure 1:**
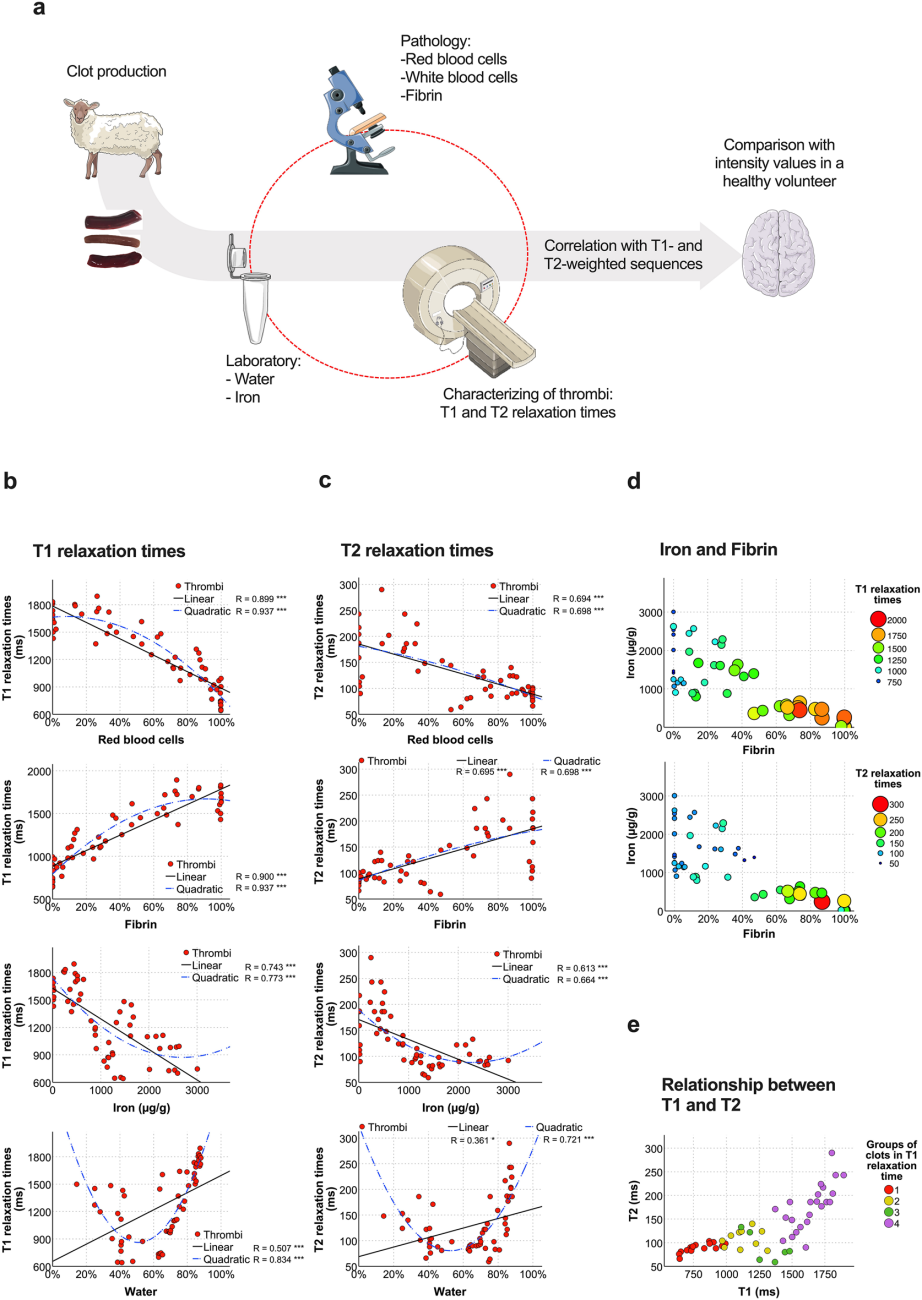
T1 and T2 relaxation times differ with histological and chemical clot composition. (**a**) Diagram of the main steps in the study and the variables evaluated. (**b**,**c**) Distribution of T1 (**b**) and T2 relaxation times (**c**) dependent on thrombus red blood cell, fibrin, iron, and water content (both in descending order). Quadratic (in blue) and linear regression models (in black) are displayed for each component with the corresponding R and P-values (P-values * < 0.01; ** < 0.001; *** < 0.0001). (**d**) Bubble plots for changes in T1 and T2 relaxation times (in descending order) dependent on iron and fibrin content. (**e**) Scatterplot comparing clot T1 and T2 relaxation time values. Grouping of thrombi by color refers to clot classification based on the T1 relaxation times from Fig. 2a. Sheep and component images in the illustration from Servier Medical Art (http://smart.servier.com).

In view of the considerable increase in imaging for diagnosis and therapy selection in acute ischemic stroke, there is an opportunity – and a need – to improve MRI sequences to enable reliable thrombus identification and profiling as an aid in selecting the potentially most successful stroke therapy in each case. The object of this study was to investigate the typical T1 and T2 relaxation times of clot analogs spanning a broad histological spectrum as a basis for understanding thrombus imaging variability in MRI. In addition to the histological characteristics, we also analyzed the effects of water content and iron content and the imaging characteristics of clots in T1- and T2- weighted sequences at a field strength of 1.5 T. Lastly, we compared the results obtained in clots with the normal grey and white matter of a test subject to identify, which type of sequence would deliver better clot contrast depending on clot histology (Fig. 1a).

## Results

### RBC content correlated more closely with iron than with water content

Median levels in the 57 clot analogs were: RBC 71.4% (IQR: 25.8, 95; range: 0, 99); fibrin 28.6% (IQR: 4.8, 74.1; range: 0, 100); iron 1067 µg/g (IQR: 412, 1625; range: 9, 3005); and water 69.8% (IQR: 42.1, 84; range: 14.3–88.1). All thrombi were well oxygenated (mean partial pressure of oxygen: 217 ± 25 mm Hg), without large differences across the histological clot spectrum (P = 0.329). Percentages of fibrin and RBC correlated inversely (Spearman ρ = − 0.996, P < 0.0001) and WBC levels decreased in clots with higher fibrin contents (Spearman ρ = 0.592, P < 0.0001). The iron content was closely correlated with the RBC content (Spearman ρ = 0.804, P < 0.0001). Correlation coefficients were lower for the water content, which correlated negatively with the percentage RBC content (Spearman ρ = − 0.583, P < 0.0001) and positively with the fibrin content (Spearman ρ = 0.576, P < 0.0001). Overall, the range of T1 relaxation time for clots was 641 to 1893 ms (median: 1253 ms, IQR: 968, 1605) and 59 to 290 ms for T2 (median: 112 ms, IQR: 90, 159). Of all the clot components, fibrin exhibited the highest correlation coefficient with T1 times (Spearman ρ = 0.919, P < 0.0001) and iron with T2 times (Spearman ρ = 0.686, P < 0.0001).

### Relaxation times differed depending on clot histological and chemical composition

Overall, changes in T1 and T2 relaxation times differed depending on clot composition, and individual clot components had different effects on T1 and T2 times. The strongest association found was between histological features (RBC and fibrin contents) and T1 times (Fig. 1b and c). T1 relaxation times could be predicted in 80% and 88% of cases based on the RBC and fibrin contents using linear and quadratic regressions, respectively (Fig. 1b). The quadratic model for fibrin predicting T1 times showed more pronounced changes when fibrin content in thrombi is less than 60% (i.e. predicted T1 for 10% fibrin: 974 ms, 30% fibrin: 1274 ms, and 50% fibrin: 1491 ms) than if exceeded the 60% (i.e. predicted T1 for 60% fibrin: 1568 ms, 80% fibrin: 1658 ms, and 100% fibrin: 1664 ms). Extended data Fig. 1 in the supplemental materials displays the prediction parameter values for these regressions. Moreover, Fig. 1d plots the changes in relaxation time with the iron and fibrin contents and Fig. 1e the relationship between T1 and T2 times.

### Decision tree for first biomarkers and relaxation times: the fibrin content in clots determines T1 times, the iron content T2 times

**Figure 2:**
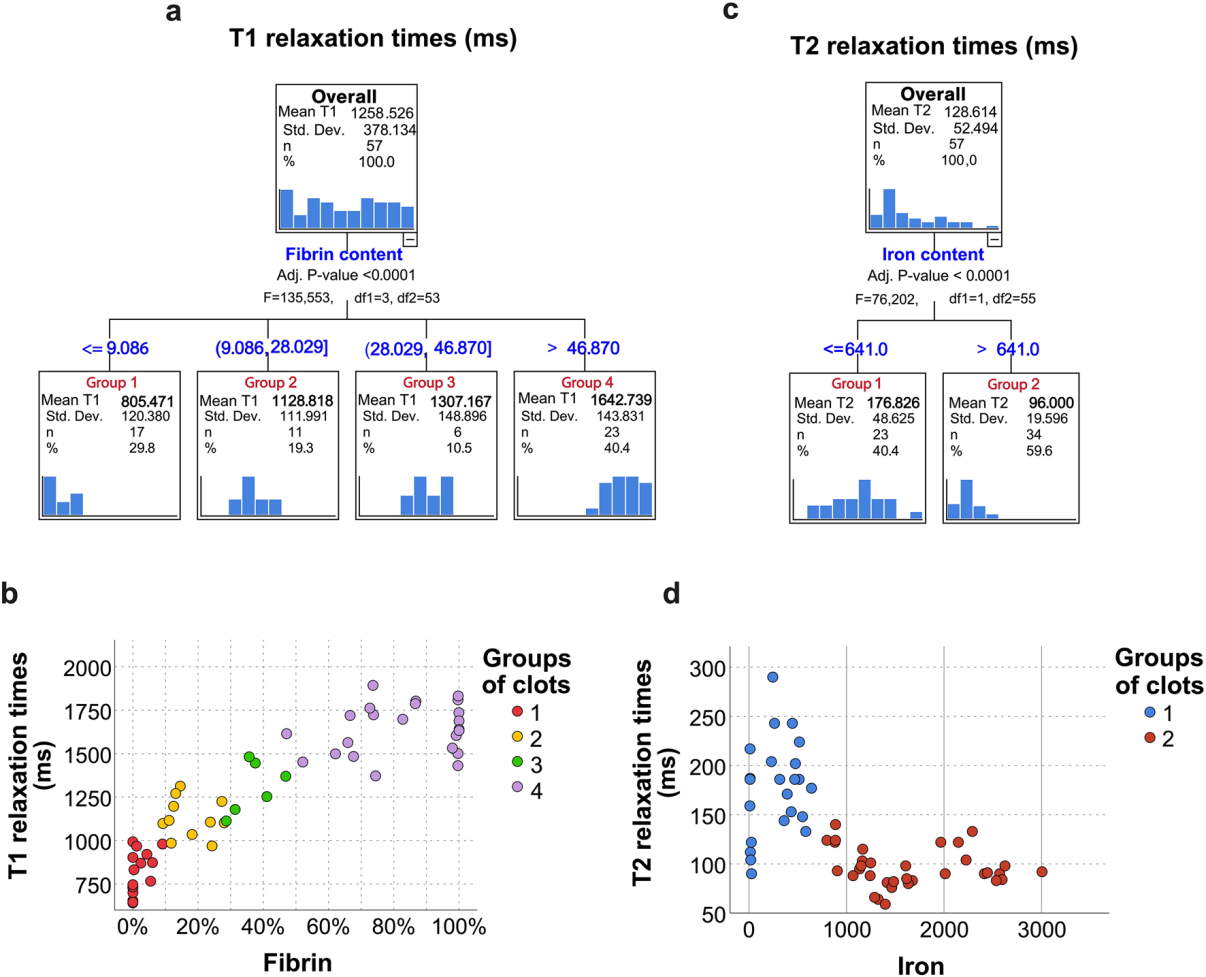
Fibrin content was the primary imaging biomarker for T1 relaxation time, raising T1 values, and the iron was the primary imaging biomarker for T2 relaxation time, inducing a T2 decrease. (**a**) Decision tree analysis results for T1 relaxation time as dependent variable, disclosing four groups of thrombi at the first level differentiable according to the effect of fibrin content on T1 times, with a high degree of significance (P < 0.00001). (**b**) Scatterplot representation of the T1 decision tree from above. The colors represent each of the newly defined clot groups. (**d**) Decision tree for T2 relaxation times, which selected iron content as the independent classifying variable (P < 0.00001); only two groups of clots were defined at the first level. (**d**) Scatterplot representation of the T2 decision tree from above.

Using a one-level decision tree, fibrin was selected as the most important clot component conditioning T1 (P < 0.0001). Four groups of clots based on fibrin content were defined based on T1 relaxation times (Fig. 2a and b): ≤ 9% fibrin (mean T1: 805.4 ms ± 120.4), between 9 and ≤ 28% (1128 ms ± 112), from 28 to ≤ 47% (1307.2 ms ± 149), and more than 47% (1642.7 ms ± 143.8) (P < 0.0001).

Using the same method for T2 relaxation times, both the RBC and iron contents had an equal effect on T2 times. The two groups the decision tree created by selecting either the RBC or iron content as the primary independent variable were equivalent (included the same thrombi) and therefore exhibited identical T2 relaxation times [group 1 (≤ 52.8% RBC or ≤ 624 µg/g iron) mean T2: 176.8 ms ± 48.6; group 2 (> 52.8% RBC or > 624 µg/g iron) mean T2: 96 ms ± 19.6, P < 0.0001]. Nevertheless, the iron content-based model had a lower cross-validation error, indicating slightly better performance than the RBC-based model (1479 ± 310 and 1971 ± 546, respectively). Therefore, this one is displayed in Fig. 2c and d.

### T1 times of fibrin-rich clots can be subclassified based on water content

Decision trees selected the following secondary imaging biomarkers for further refinement of the primary biomarker groups for each T1 and T2 relaxation time. Differences in percentage water content significantly influenced the T1 relaxation times, but only in thrombi with more than 47% fibrin (P = 0.002) [supplemental materias: extended Data Fig. 2 (part 1)a, c, and d]. The RBC-rich thrombi (group 1, fibrin < 9%) could also be classified into a further two “very low” fibrin content subgroups (P = 0.048) [supplemental materials: extended Data Fig. 2 (part 1)a, b, and d].

### Iron concentration was selected as second biomarker for T2 relaxation in RBC-rich clots

The effect of iron concentration was to greatly shorten T2 times up to a concentration of 641 µg/g, after which the changes in T2 times with further increases in iron content were less marked. Even so, iron content was re-selected to further characterize thrombi containing more than 641 µg/g of iron (P < 0.0001) [supplemental materials: Extended Data Fig. 2 (part 2)a, c, and c].

### MRI sequences: T2w DRIVE followed by T1w turbo IR images provide the widest range of signal intensities for differentiating thrombi

MRI sequence type and parameters and the normalization method used (intensity values from 0 to 1 for T1w TSE and T2w DRIVE and from − 1 to 1 for T1w IR images) are described in the Methods section. Unless otherwise specified, we grouped the clots for further analysis using the T1 classification tree for primary imaging biomarkers.

**Figure 3:**
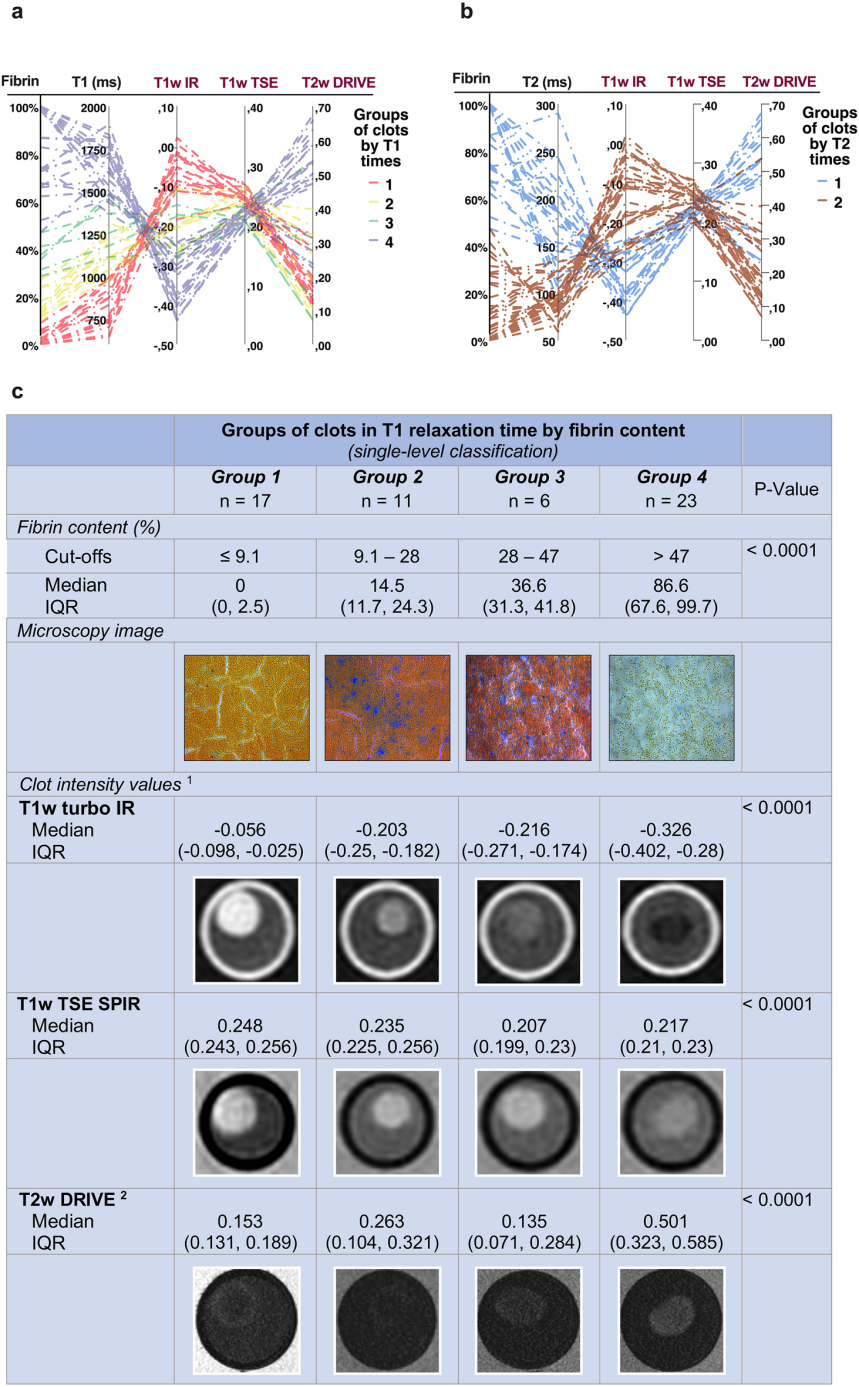
The clot histological spectrum shows the broadest range of possible intensity values in T2w DRIVE images followed by T1w inversion recovery images. (**a**) Parallel coordinates in the graph display the normalized intensity scales of the applied imaging sequences, and across the scales, in broken lines, each of the 57 thrombi by T1 classification group (single level) in different colors. (**b**) Likewise, graph with parallel coordinates plot displaying the T2 classification (single level). (**c**) Schematic overview of the T1 relaxation time clot groups, their neuropathological features, and imaging examples. Groups (or nodes) are in ascending order with regard to fibrin content. Results are expressed as medians and interquartile ranges (IQR). ^1^The range of normalized intensity values for the MRI sequences is from zero to 1 for T1w TSE SPIR and T2w DRIVE, and from − 1 to 1 for T1w turbo IR. ^2^For display purposes, the intensity window of T2w DRIVE group 1 was changed manually to allow better visualization. ^3^P-Values: Kruskal–Wallis Test. T1w Turbo-IR: axial T1-weighted turbo-inversion-recovery; T1w TSE SPIR: axial T1-weighted turbo spin-echo with fat suppression using Spectral Presaturation with Inversion Recovery; T2w DRIVE: axial T2-weighted turbo spin-echo with DRIVen Equilibrium magnetization refocusing.

**Figure 4:**
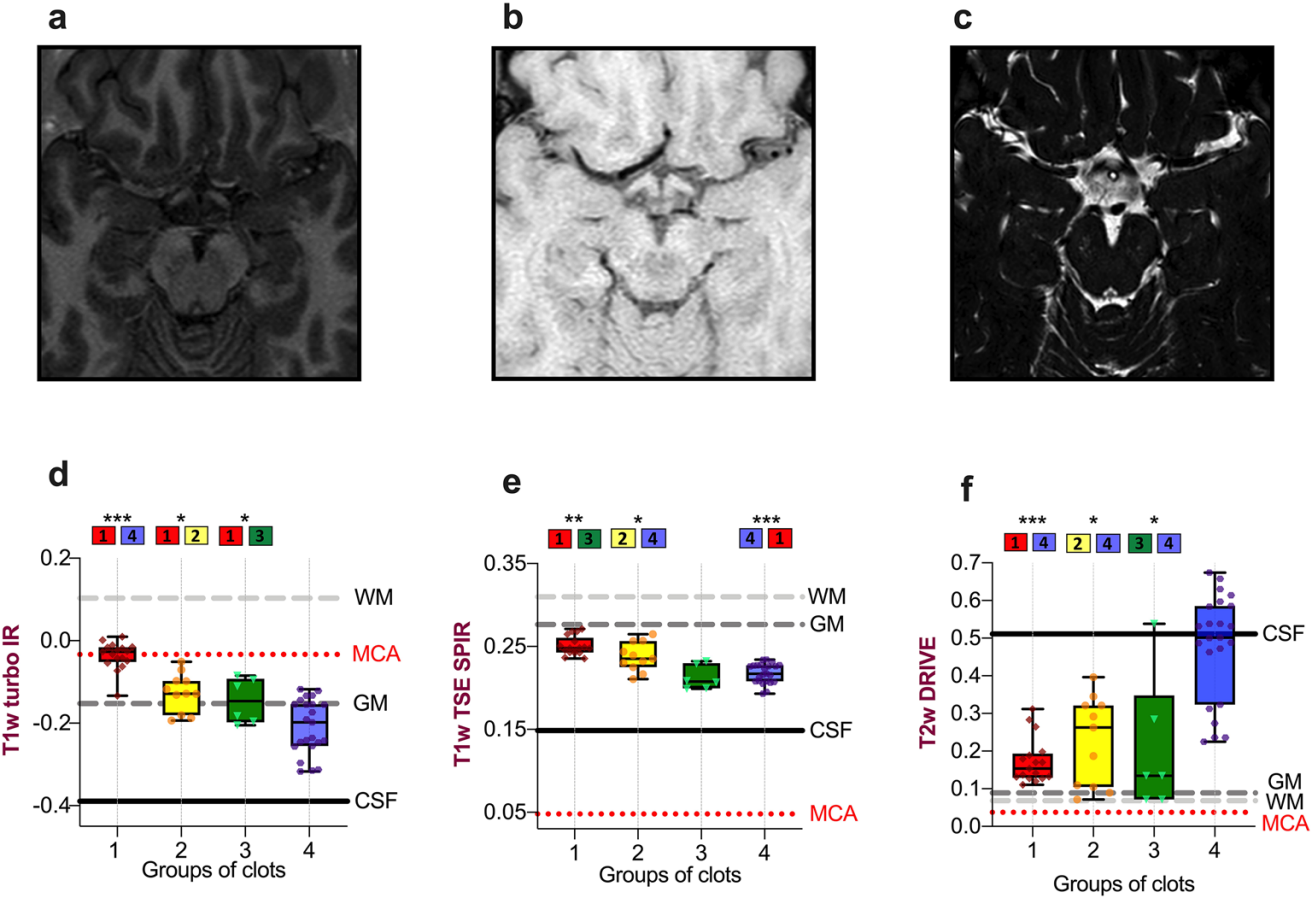
On T1w TSE images all clot types display higher intensity than the MCA and CSF, facilitating clot contrast, especially RBC-rich thrombi; and on T2w DRIVE images, clots with high fibrin content are distinguished by a higher signal. (**a**–**c**) Examples of the sequences performed on the test subject using the same MRI scanner and equivalent MRI parameters as in the clot imaging. (**a**) Axial T1-weighted turbo Inversion Recovery (T1w Turbo IR). (**b**) Axial T1-weighted turbo spin-echo with fat suppression using Spectral Presaturation with Inversion Recovery (T1w TSE SPIR). (**c**) Axial T2-weighted turbo spin-echo with DRIVen Equilibrium magnetization refocusing (T2w DRIVE). (**d**–**f**) Plots of the four clot groups defined by the fibrin content effect on T1 relaxation times (single-level decision tree, see Fig. 2) by clot signal intensity in each sequence. Reference values: the test subject's white matter (WM), grey matter (GM), cerebrospinal fluid (CSF), and middle cerebral artery (MCA). (**d**) Boxplot of the T1 clot groups and their intensity signal in T1w turbo IR images. The pairwise comparison showed significant differences in clot intensity in T1w IR between group 4 (fibrin content > 47%) and groups 1 (< 9% fibrin, P < 0.0001), group 2 (9–28% fibrin, P = 0.034), and group 3 (28%–47% fibrin, P = 0.032). (**e**) Boxplot of the T1 clot groups and their corresponding intensity values in T1w TSE SPIR. There were appreciable signal differences between group 1 and groups 3 (P < 0.001) and 4 (P < 0.0001) and between groups 2 and 4 (P = 0.026). (**f**) Boxplot of clot groups on T1 times by observed T2w DRIVE signal intensity. Clot analogs in group 4 showed significantly different intensities than the other groups [group 1 (P < 0.0001); group 2 (P = 0.003); group 3 (P = 0.006)].

The parallel coordinates plot in Fig. 3a shows the distribution of the T1 clot groups on the intensity scales for the different MRI sequences used. The T2w images provided the broadest range of intensity values for the clot spectrum (median: 0.274; IQR: 0.137, 0.487; range: 0.071, 0.674), followed by the IR images (median: − 0.131; IQR: − 0.193, − 0.054; range: − 0.317, 0.009), and the T1w TSE with the smallest range (median: 0.230, IQR: 0.215, 0.245 range: 0.193, 0.271). Groups 1 (RBC-rich clots) and 4 (fibrin-rich clots) can be found at the two ends of the clot intensity range on the IR intensity scale, leading to significant differences in imaging (Fig. 3a and c). In contrast, clot histologies converged around a narrow range of T1w TSE intensities. On T2w DRIVE, most of the RBC-rich clots (group 1) lay in a range of higher intensities, separate from the intensity range of other clot groups, grouped at lower T2w intensity values. The parallel coordinates plot for the T2 time clot classification shows a wide intensity range in T2w DRIVE images of thrombi with regard to the iron content (Fig. 3b). In this sense, Fig. 3c shows that in T1w IR images the signal intensity of RBC-rich thrombi (group 1) was the opposite of that of the fibrin-rich clots (group 4) (P < 0.0001), where fibrin-rich clots displayed lower intensity (darker) than RBC-rich thrombi (brighter). In contrast, signal intensity increased with fibrin content in clots in the T2w DRIVE sequence (P < 0.0001). In summary, we found that fibrin rich clots had the longest relaxation times in T2 and in T1, leading to high signal intensity in the T2w DRIVE sequence, and in turn, RBC rich clots had the shortest relaxation times and yielded higher intensity in T1w sequences [supplemental materials: extended Data Fig. 3a–f]. Besides, although there were statistically significant differences in the intensity values for the different clot groups in each sequence, pairwise comparison had difficulty in discriminating between groups 2 (9–28% fibrin) and 3 (28–47% fibrin) in all sequences (all P-values > 0.08 Bonferroni adjusted, see Fig. 4d–f).

### Regardless of their histology all clots are distinguishable from a patent artery in T1w TSE images

To translate our results into actual practice, we exploratively compared normalized clot intensities with the normalized brain signal intensities of grey matter (GM), white matter (WM), cerebrospinal fluid (CSF), and the middle cerebral artery (MCA) of a test subject. Figure 4 summarizes these results.

In T1w IR (Fig. 4a and d), the average intensity of a patent MCA was in-between the signals for WM and GM, distinguishable from the CSF, which exhibited the lowest intensities. Nevertheless, the vast majority of clots showed intensities below the MCA, falling in an intensity range similar to that of GM and thus limiting the ability to identify clots in a real setting with the IR sequence used here.

On the other hand, in T1w TSE images (Fig. 4b and e) the signal of a patent MCA is low, darker than the surrounding CSF and considerably below GM and WM signal intensities. Here, all clot groups, regardless of their histology, entail a notable increase in the signal intensity of the MCA, but they not overcome the high signal intensity of brain parenchyma (Fig. 4b). Although the higher the intensity of thrombi on T1w TSE sequences, the lower the fibrin content, the possibility of discriminating among all clot histologies might be limited because of the narrow intensity range in which all four groups are located (Fig. 4e).

### T2w DRIVE images readily detect thrombi of > 47% fibrin content, the more fibrin, the greater clot hyperintensity

In the T2w DRIVE sequence, arteries and brain parenchyma have very low signal intensities, in contrast to CSF, which is hyperintense (Fig. 4c and f). Only a few thrombi with very high iron content may not be distinguishable from the neighboring GM or MCA in T2w DRIVE sequences, though most should exhibit higher signals than the MCA proper. Notably, the highest signal intensities were recorded for thrombi with more than 47% fibrin which, in some cases, could even reach the signal intensity of the CSF (group 4, Fig. 4f).

## Discussion

Our experimental study has demonstrated that acute stroke MRI protocols for visualizing acute ischemic stroke thrombi can be improved. The broad spectrum of clot analog compositions examined produced T1 relaxation times ranging between 641 and 1893 ms and T2 times between 59 and 290 ms^10,20–22^. The fibrin and iron contents of thrombi were the primary imaging biomarkers and determined the T1 and T2 relaxation times of clots, respectively. Translated into MRI imaging sequences, acute ischemic stroke thrombi should have a higher intensity signal than the surrounding structures in both T1w TSE and T2w DRIVE sequences, though indicating opposite clot compositions: hyperintensity in T1w TSE images indicates high RBC content and in T2w DRIVE images high fibrin content.

### T1 relaxation time

In line with the results of regression models, the decision tree selected fibrin content as the first biomarker for T1 relaxation times, defining up to four clot groups, and water content as a secondary biomarker, allowing further differentiation among fibrin-rich clot analogs.

There is no simple explanation for modulation of T1 relaxation times by thrombus fibrin content. One possibility could be that fibrin content is a good marker of clot water content and that a larger amount of free water may act as a factor lengthening relaxation times^9^. However, in our measurements the relationship between water and relaxation times was not linear but parabolic, with the highest T1 values at the low and at the high-water content, and a minimum T1 at 50% water (Fig. 1b). A similar curve model based on osmolarity variations in cells has been reported by Raaphorst et al.^12^. Thus, T1 relaxation times seems to be sensitive to any change in the relative size of the different water compartments. Only limited data on water content and variations on clot histologies are available. During clot retraction, which involves water loss by serum expulsion, T1^23^ and T2 relaxation times decrease^11^, though no significant changes in T1 relaxation times after clot retraction have also been described^10^. This could mean that other factors besides gross water content differences in thrombi make a larger contribution to the T1 relaxation time. For example, it has been proposed that a thrombus protein gradient may produce heterogeneous clot images^10^. Although protein content was not directly addressed in this study, previous studies estimated proteins in packed erythrocytes at 30% (300 g/kg)^8^ similar to fibrin fibers, which ranged between 20 and 28% (280 g/kg)^24^. Therefore, the possibility that variations in clot protein concentration depending on the predominant histological clot component may induce distinctive clot signal intensities can most probably be ruled out.

Fibrin fibers in human brain tissue have a rather diamagnetic susceptibility that does not differ much from water or other tissues in general^25^. Nevertheless, changes in thrombi from a more paramagnetic (RBC component) to a more diamagnetic susceptibility have been detected when fibrin fibers build up as the RBC content declines, resulting in lengthening of both T1 and T2 relaxation times^26^. In addition, it is important to note that fibrin structure is variable. Thrombi with thicker fibers exhibit higher permeability and porosity and are associated with higher rates of fibrinolysis than dense, finely packed fibers^27,28^. Furthermore, parallel packing of fibers, which seems to occur in thrombi formed in flow conditions, exhibits greater permeability than thrombi with randomly oriented fibers^27,29^. It is still unclear to what degree porosity and permeability of clots influence relaxation times. However, according to studies in confined systems, large pores should be related to higher T1 relaxation times and small pores to shorter times^30,31^. Thus, the fibrin structure at a constant RBC content may be a fundamental factor determining clot permeability and porosity and affecting T1 times. In summary, all these aspects indicate that the fibrin contribution to T1 relaxation times at 1.5 T might be based on the positive association of fibrin with water content, inversely correlated with the RBC content, and on differences produced by fibrin structure and density, modifying clot permeability and pore size, modulating in this way the water mobility.

### T2 relaxation times

Based on the regression model results, the relationship between histological clot attributes and T2 relaxation times was more random than for T1 times. The decision tree selected Fe III content to be more accurate than the RBC content for defining two principal groups of thrombi on the basis of T2 relaxation (P < 0.0001). Many researchers have reported that hematocrit values, an indirect measure of iron, make a significant contribution to reducing T2 relaxation times^10,32,33^. Paramagnetic Fe III is generally attributed to methemoglobin, present in oxygenated blood at very low concentrations^18^. In experimental thrombi, rate of methemoglobin formation is very low (0.042%/hour over 30 h)^16^ with a concentration peak on day seven^22^. Thus, the methemoglobin might not be the sole source of Fe III in 24 h-old clot analogs with ample oxygenation levels. A possible explanation considers also oxyhemoglobin, which has been reported to contain Fe II and between 50 and 70% Fe III^18,34^. Although the rate at which these clot compounds build up in the RBCs in a thrombus is unclear, it is plausible to assume that the iron measured in our study might be a combination of oxy and methemoglobin. Furthermore, it is important to note that clots with high fibrin content uniformly had low iron content. In contrast, RBC-rich clots (with low fibrin content) exhibited varying levels of iron (refer to Fig. 1c). This finding supports the decision tree's use of iron, rather than RBC content, to predict T2 relaxation times.

### MRI sequences

Judging from the MRI sequences examined, T2w DRIVE images can readily detect mixed to fibrin-rich thrombi capable of attaining the very high-intensity values of CSF in some cases. Only a few RBC-rich thrombi with pronounced shortening of T2 relaxation time due to high iron content may have very low signal intensity and be indistinguishable from the patent MCA^10^.

With T1w TSE SPIR imaging, all types of clots should be strongly hyperintense, and therefore, easily recognizable from CSF or the patent MCA. Previous studies show that the inherent low blood signal of non-contrast T1w TSE images facilitates cerebral venous thrombosis detection^35^. Our results reveal that the more hyperintense a clot is in T1w images, the greater the RBC content. Nevertheless, the range of possible signal intensities to discriminate between clot histologies is very narrow (Fig. 3a). It should be noted that all of our "acute" clot analogs had a higher signal intensity than the patent MCA and CSF, but lower than the GM. However, it is very likely that the signal intensity of thrombi in T1w TSE images increases over time due to increased methemoglobin conversion, as shown in previous studies dealing with subacute venous thrombosis^22,36^. This aspect might be useful for distinguishing between acute and subacute intracranial thrombi. Finally, in T1w turbo IR all types of thrombi yield a more hypointense (darker) image than the patent MCA, which could pose a challenge for clot identification. However, though not examined in this study and not common in IR imaging, gray scale inversion of the IR image could overcome this limitation of the human eye.

### Limitations

Although clinical laboratory results are normally quite precise, the minimal sample size needed to obtain the most reliable analysis results is unclear. Some error variability is inherent to MRI measurements due to effects unaccounted for when determining relaxation times using the MIXED sequence, slight variations in sequence imaging parameters, inhomogeneous display of imaging signals within the ROIs evaluated, and practical assumptions for normalizing signal intensities. Using a coarse model seems justified, because higher accuracy and precision does not add information or alter the conclusion of this study, as real clots and real brain tissue exhibit a broad range of signal intensity-determining features, i.e. a broad range of relaxation times, possibly even multi-exponential relaxation. Even seemingly precise values would not allow direct inferences for optimal imaging protocols in the clinical setting. Additionally, it is important to note that potential MRI artifacts that may affect vascular imaging in these sequences have not been assessed. Still, the findings presented here may help in optimizing the clinical imaging protocols used in stroke diagnosis for improved clot detectability and characterization.

## Conclusion

The fibrin and iron contents were the primary imaging biomarkers for clot analogs, determining their T1 and T2 relaxation times, respectively. Two MRI sequences, T1w TSE and T2w DRIVE, are capable of showing the vast majority of clots as hyperintense with good clot image contrast. Particularly for RBC-rich thrombi, T1w TSE signal intensity increases with the RBC content. On the other hand, mixed to fibrin-rich thrombi are better visualized in T2w DRIVE according to the principle of the more fibrin, the higher the intensity. Accordingly, MRI stroke protocols that include these sequences might display the full spectrum of human stroke thrombi as hyperintense structures. Determining clot histology before initiating thrombectomy is essential, since in situ clot removal depends on the histological nature of each clot. Thus, where clots can be visualized distinctly and characterized histologically, patients will benefit from personalized stroke therapy decided at the point of diagnostic imaging based on improved MRI imaging protocols.

## Methods

### Study design

In this experimental, proof-of-principle study of human-like acute ischemic stroke thrombi, we investigated tissue-specific MRI parameters of thrombi in their broad histological spectrum to subsequently determine clot composition-based image performance of T1 and T2 weighted sequences. Fifty-seven clot analogs of different but predefined composition were imaged with a 1.5 T MRI scanner (Philips Ingenia 1.5 T, Philips, Best, NL) at 24 h from production in six different sessions. Previous results of clot analog imaging at 24 h by means of CT have previously been published elsewhere^37^. Thrombi were produced by Cerenovus (Mechanical and Industrial Engineering Department of the Galway-Mayo Institute of Technology, Galway, Ireland)^38^. Thrombus material from each of the clots examined by MRI was preserved for neuropathological analysis [red blood cells (RBC), white blood cells (WBC), and fibrin] and laboratory analysis (water and iron content). Clot T1 and T2 relaxation times were determined along with the image characteristics of three sequences analyzed: a T1-weighted Inversion-recovery sequence (T1w IR), a T1-weighted turbo spin echo with fat suppression (T1w TSE SPIR), and a T2-weighted 3D TSE with magnetization refocusing to remove T1 dependence (T2w TSE DRIVE). Two of the authors (AVG senior neuroradiologist, and HK, senior medical MR physicist) were in charge of clot measurements. Finally, we repeated the same sequences used for the clots on a test subject to determine the normal intensities and exploratively determine which sequence could deliver better clot contrast against brain tissue. A detailed explanation appears in the MRI section below. Figure 1a summarizes the main steps in the study. This study was approved by the University of Münster, Germany. Clot extraction from ovine species was performed in accordance with relevant guidelines and regulations.

### Clot production

Venous blood was obtained from the jugular vein of ovine species at a licensed facility (Ash Stream Ltd., Mayo, Ireland) with trained veterinarians and avoiding animal suffering. Blood was collected from the jugular vein using a syringe pre-loaded with citrate-based CPDA anticoagulant and dispensed into a container open to the air at ambient temperature. The blood was transported at ambient temperature in sealed containers and then prepared at ambient temperature open to the air in the laboratory. Thrombi were produced from ovine venous blood by Cerenovus (Galway, Ireland). Thrombus production used methods previously described to create thrombi with different red blood cell (RBC) fractions from blood mixtures with hematocrit levels of 84%, 20%, 5%, 1% and 0%^37,38^. The thrombi were matured using two methods, platelet-driven contraction and manual contraction using a centrifuge (with the 84%–hematocrit clot omitted from this latter group). The blood was clotted in sealed containers at 37 °C using calcium chloride within six hours of blood collection from the donor. Approximately 16 h after clotting the thrombi were manually contracted (when appropriate), weighed, and cut to size at ambient temperature in the open air. The thrombi were transported and stored in their own serum in sealed containers at approximately 3 °C until use.

### Histological analysis

Thrombus material from each of the clots examined by MRI was immediately fixed in 10-% phosphate-buffered formalin. The formalin-fixed clots were embedded in paraffin, cut into 5-µm thick slices, and stained with hematoxylin and eosin and Martius Scarlet Blue (differentiating fibrin and RBCs) (2). Two of the authors (AJ, senior neuropathologist, and AVG, neuroradiologist) carried out the histological evaluation. Using × 40 magnification, clot cores were photographed (Olympus BX43 microscope + digital camera). ImageJ software (version 1.52a) was used for the quantitative threshold-based automatic measurement of the percentage fibrin, RBCs, and WBCs.

### Clinical laboratory analysis

The water content was determined by drying the samples at 90 °C using the standard protocol of removal of moisture at 90 °C for 48 h until complete dryness in a drying cabinet and then measuring weight loss with an analytical balance. Iron (III) was measured by spectrometry. To determine the water content, samples were weighed (ME235P, Sartorius, Göttingen, Germany) before and after drying at 90 °C for three days. For the iron determination samples were dried at 70 °C overnight to reach a constant mass and transferred to a 500-μl PFA vessel (AHF-Analysetechnik, Tübingen, Germany). An amount of 100 μl of HNO_3_ (60% v/v) was added and incubated overnight at room temperature. Before measurement, samples were incubated at 70 °C for 90 min, allowed to cool, and diluted tenfold with H_2_O. Next, 10 μl of the solution was placed directly in a graphite furnace for atomic absorption spectrometry (AA6300, Shimadzu, Kyoto, Japan). For each determination, measurements were carried out in triplicate. The recommended operating conditions for the spectrometer were: hollow-cathode lamp current: 12 mA; absorption wavelength: 248.3 nm; BGC-D2-Modus; gap width: 0.2 nm. Working solutions containing 0, 5, 10, 15, and 20 µg/l FeCl_3_ were used as standard solutions. The Limit of Quantitation, Limit of Detection, and the linear range for Fe analysis were 0.5 µg/l, 0.1 µg/l, and 0.5–20 µg/l, respectively. The intra and inter-day precision of the assay, expressed as a coefficient of variation, ranged from 2.8 to 6.5%. For determination of partial pressure of oxygen (pO2), 10 mg clot tissue and a 1-cm dry ice (3 mm pellets) layer was placed in a mill grinding chamber (TUBE MILL CONTROL, IKA Werke, Staufen, Germany). After homogenization (2 min) the sample was transferred into an Eppendorf tube and 200 µl PBS buffer was added, followed by centrifugation at 13,000 rpm. The samples were analyzed using a commercial blood gas analyzer (ABL800 FLEX, Radiometer, Copenhagen, Denmark).

### Phantom

As cylindrical structures, thrombi were positioned parallel to the long axis of the Eppendorf tubes and fixed centrally in 2-% agarose, carefully avoiding air bubbles at the interface. For control and reference purposes, two further Eppendorf tubes were added to the experiment: one with plain 2-% agarose and one with MRI blood mimicking fluid (Shelley Medical Imaging Technologies, North York, Canada). For imaging, all tubes were placed in a plastic tube rack, which was immersed in a plastic container filled with 2.5 L of water + 0.2 mL gadolinium contrast agent (Gadovist^®^) within a plexiglass holder at room temperature. The phantom was positioned inside a 20-channel (standard) head coil. In each of the six sessions, different clot histological types were imaged to facilitate the in-session comparison of clot features.

### MRI measurements

To check whether different clot compositions exhibit different appearances on MR imaging, (1) clot relaxation times were measured to ascertain to which extent they are determined by clot composition, and (2) the appearance of the clots was tested in three basic sequences. Two of the authors (AVG senior neuroradiologist, and HK, senior medical MR physicist) were in charge of clot measurements.

Clot T1 and T2 relaxation times were determined using the MIXED sequence, which interleaves a multi echo IR with a Spin Echo (SE) sequence, allowing simultaneous determination of T1 and T2 times in a single measurement using the recursive least squares method (RLSQ)^39^ with the following parameters: single slice acquisition, thickness 3 mm, axial to the Eppendorf tubes, FOV 180 mm, voxel size 1.4 × 1.7 mm; repetition time (TR) for the SE part 1000 ms, TR for the IR part 2260 ms, inversion time (TI) 500 ms, echo times (TE) 13 ms and 100 ms, performed as a Turbo sequence with an echo train length (ETL) of 10. Relaxation times were displayed as maps as calculated by the scanner software assuming monoexponential relaxation.

To explore whether the different signal intensities and contrasts of clot analogs represent the range of T1 and T2 relaxation times in clinical imaging, two different T1-weighted and one T2-weighted sequences were selected from brain imaging protocols available for the scanner and used in routine imaging: a T1-weighted turbo-inversion-recovery sequence (T1w Turbo-IR), a T1-weighted TSE sequence with spectral fat suppression using Spectral Presaturation with Inversion Recovery (T1w TSE SPIR), and a 3-dimensional T2-weighted TSE sequence with 'driven equilibrium' reset pulse, i.e., magnetization recovery to remove dependence on T1 for tissues with long T1 times (T2w TSE DRIVE, DRIven Equilibrium), all without specific contrast optimization for clot imaging.

The rationale for sequence selection was as follows: T1w and T2w images are commonly used in brain imaging because they allow visualization of brain lesions. Proton density weighted imaging is less common. Here, in addition to 'plain' T1w and T2w imaging, a T1w inversion recovery sequence was employed, which exhibits a less simple dependence on T1 relaxation. In contrast to the other two sequences, which are displayed as positive signal intensities, an IR sequence is commonly displayed with positive and negative signal intensities, with zero intensity (= absence of signal) being displayed as medium gray.

Acquisition was performed in coronal orientation (cutting orthogonally through the Eppendorf tubes), and imaged based parallel imaging (SENSE) was chosen with a factor of 2. Other relevant parameters are given in supplementary information (Table 1). To calculate clot relaxation values and obtain clot image signal intensity values, circular ROIs with a minimum area of 5 mm^2^ were drawn using the MR scanner's user interface measurement tools on the relaxation time maps generated on a representative slice orthogonally intersecting the tubes, taking the mean over the ROI. To compare clot intensities with intensity values of normal grey matter (GM), white matter (WM), cerebrospinal fluid (CSF), and the middle cerebral artery (MCA), the sequences were repeated on a test subject. All intensity values of the clots and the brain tissue of the test subject were recorded.

### Normalization of intensity values

To compare signal intensities across different imaging sessions, which usually exhibit different intensity scaling due to, e.g. coil load differences, differences in power optimization, and gain settings, the intensity values must be converted to a common scale. In this study a scale of from 0 (absence of signal) to + 1 (full signal of pure water) for unsigned (positive only) intensities (T1w and T2w TSE sequences) was selected, defining an intensity of 1 as water in the absence of signal attenuation due to relaxation or saturation, i.e. assumed values of TE = 0 and TR = infinite. For the T1w IR sequence with signed signal intensities, a scale of from − 1 (fully inverted signal of pure water) to + 1 (full signal of pure water) was selected.

The rescaling factor was determined by comparing the calculated signal intensity of a reference sample of pure agar for the three sequences in each session based on measured T1 and T2 values of the sample prepared for that session with the measured intensity value of that sample in each sequence. This factor was then applied to every measured intensity value in the respective sequence.

In the measurement taken using the volunteer, the gray matter of the right temporal lobe was used as reference for the rescaling factor.

In the two scales, signal intensities I of the Agar reference sample in each session, and of the gray matter serving as reference in the volunteer measurement, were calculated as follows:

For the T1w and T2w sequences (unsigned, T1w TSE SPIR and 3D T2w TSE DRIVE)):

1 $${\text{I }} = {\text{ PD }} \times \, ({1} - {\text{e}}^{{ - {\text{TR}}/{\text{T1}}}} ) \, \times {\text{ e}}^{{ - {\text{TE}}/{\text{T2}}}} \times {\text{ sin}}\left( {{\text{FA}}} \right),$$

and for the IR sequence (signed, T1w Turbo-IR):

2 $${\text{I }} = {\text{PD }} \times \, \left( {{1} - {2 } \times {\text{ e}}^{{ - {\text{TI}}/{\text{T1}}}} + {\text{ e}}^{{ - {\text{TR}}/{\text{T1}}}} } \right) \, \times {\text{ e}}^{{ - {\text{TE}}/{\text{T2}}}} \times {\text{ sin}}\left( {{\text{FA}}} \right),$$

with PD assumed to be 1 for Agar, and 0.85 for gray matter. The FA (flip angle) was taken as 90° assuming classical Spin-Echo imaging. To allow for the use of turbo sequences, instead of nominal TR the actual TR was taken as the distance from the last refocusing inversion pulse to the following excitation, i.e. TR_act_ = TR_nom_–ETL × Echo spacing, as z magnetization builds up only after these pulses due to saturation of magnetization by refocusing pulses.

The effect of magnetization restoration ('DRIVE') in the T2w sequence was modeled by a applying a long TR of 10 s. Side effects of spectroscopic fat suppression (SPIR), pulse imperfections, magnetization transfer, etc. were neglected.

### Statistical analysis

All statistical analyses were performed using SPSS version 25/2017 software (IBM, Armonk, NY, USA). P values ≤ 0.05 were deemed to indicate a statistically significant difference. All reported P values were two-sided. Continuous variables have been described in terms of the mean, standard deviation (SD), median, interquartile range (IQR), and range. The Kruskal Wallis test was used to test the differences in the continuous variables among more than two groups. Subsequent Mann–Whitney *U* tests identified pairwise differences between subgroups. The Spearman correlation coefficient (Spearman ρ) and the linear and quadratic regression models were used to determine the relationships between clot components and relaxation times. Results have been presented using the coefficient of determination (R^2^) and the regression coefficients (β). Decision trees were fitted using the CHAID (Chi-square Automatic Interaction Detector) algorithm to determine the most relevant clot component influencing the T1 and T2 relaxation times as well as the clot classifications based on these tissue-specific parameters. We evaluated various options for developing the decision trees, including the maximum number of groups (or nodes) or the minimum child size (minimum number of clots within a group) to present the most reliable results with the least training data and cross-validation errors. The settings of the decision trees were: maximum depth: one (for the primary biomarkers for relaxation time) or automatic (for the secondary biomarkers for relaxation time), minimum parent size: 10, minimum child size: 6, tenfold cross-validation, alpha = 0.05, Bonferroni adjusted.

### Supplementary Information

Supplementary Information.

## Data Availability

The datasets generated and/or analysed during the current study are not publicly available due to ongoing work, but are available from the corresponding author on reasonable request.

## Abbreviations

AIS: Acute ischemic stroke
NCCT: Non-contrast CT
ROI: Region of interest
HU: Hounsfield units
RBC: Red blood cells
WBC: White blood cells
ms: Milliseconds
TSE: Turbo spin eco
T1w Turbo-IR: T1-weighted turbo-inversion-recovery sequence
T1w TSE SPIR: T1-weighted turbo spin echo with frequency-based fat suppression
TSE: Turbo spin echo
T2w TSE DRIVE: T2-weighted turbo spin echo with magnetization reset pulse
SE: SPIN echo
TR: Repetition time
TI: Inversion time
TE: Echo time
ETL: Echo train length
NSA: Number of signal averages
FOV: Field of view
GM: Grey matter
WM: White matter
CSF: Cerebrospinal fluid
MCA: Middle cerebral artery
