# Supplementary material for: Making acute ischemic stroke thrombi visible in MRI imaging
Source: Sci Rep. 2024 May 29;14:12325. doi: 10.1038/s41598-024-62985-1 (PMC11137124; doi:10.1038/s41598-024-62985-1)
Supplement: Supplementary file 1 — Supplementary Information. [file 41598_2024_62985_MOESM1_ESM.docx]

**Supplementary Information**

**Making acute ischemic stroke thrombi visible in MRI imaging**

Original Research

Aglaé Velasco Gonzalez, Boris Buerke, Dennis Görlich, Cristina Sauerland, Manfred Fobker, Astrid Jeibmann, Walter Heindel, Andreas Faldum, Werner Paulus, Harald Kugel

**Correspondence to:** PhD. Dr. med. Aglaé Velasco González, Clinic for Radiology, Neuroradiology, University and University Hospital of Muenster. Albert-Schweitzer-Campus 1, Building A1, 48149 Muenster (Germany).

Email: velascoa@uni-muenster.de

**Supplementary Information**

**Table 1. *MRI sequence parameters for clot analog imaging***

| MRI sequence name | | | |
| --- | --- | --- | --- |
| **MRI parameter** | T1w TSE SPIR | T1w Turbo-IR | 3D T2w TSE Drive |
| Repetition time TR [ms] | 496 – 580 | 2303 – 1941 | 1500 |
| Echo time TE [ms] | 16 | 14 | 200 |
| Inversion time TI [ms] | - | 400 | - |
| Echo train length ETL | 7 | 5 | 50 |
| In-plane resolution  [mm x mm] | 0.8 x 0.8 | 0.8 x 0.8 | 0.2 x 0.2 |
| Slice thickness^1^ [mm] | 3 or 2 | 2.5 | 2 |
| Slice orientation^2^ | Coronal | Coronal | Coronal |
| Number of signal averages NSA^1^ | 2 or 4 | 1 | 1 or 2 |

^1^ Signal intensity and image contrast are not affected by small differences in slice thickness or signal averages. ^2^ Acquisition in coronal orientation, i.e., orthogonally to the Eppendorf tubes and therefore to the longitudinal axes of the thrombi. T1w Turbo-IR: T1-weighted turbo-inversion-recovery sequence. T1w TSE SPIR: T1-weighted turbo spin echo with fat suppression using Spectral Presaturation inversion recovery. 3D T2w TSE DRIVE: T2-weighted turbo spin echo with DRIven Equilibrium magnetization refocusing.

**Extended Data Fig. 1**

**a**

| T1 relaxation times | | | | |
| --- | --- | --- | --- | --- |
|  | % RBCs | % Fibrin | Iron (µg/g) | % Water |
| *Linear model* | | | | |
| R^2^ | 0.809 *** | 0.809 *** | 0.552 *** | 0.257 *** |
| β_0_ (SE) | 1785.902  (41) | 887.908  (32.8) | 1626.021  (56) | 654.652  (145.19) |
| β_1_ (SE) | -8.997  (0.59) | 8.984  (0.58) | -0.333  (0.041) | 9.396  (2.155) |
| *Quadratic model* | | | | |
| R^2^ | 0.878 *** | 0.879 *** | 0.598 *** | 0.695*** |
| β_0_ (SE) | 1663.63  (39.8) | 792.684  (31.5) | 1739.614  (70.3) | 2615.01  (241.5) |
| β_1_ (SE) | 1.771  (2) | 19.223  (1.9) | -0.644  (0.13) | -68.865  (8.99) |
| β_2_ (SE) | -0.105  (0.019) | -0.105  (0.019) | 0.00012  (4.8E-5) | 0.676  (0.077) |

**b**

| T2 relaxation times | | | | |
| --- | --- | --- | --- | --- |
|  | % RBCs | % Fibrin | Iron (mg/g) | % Water |
| *Linear model* | | | | |
| R^2^ | 0.482 *** | 0.483 *** | 0.376 *** | 0.130 * |
| β_0_ (SE) | 185.142  (9.4) | 88.881  (7.49) | 170.721  (9.18) | 68.959  (21.8) |
| β_1_ (SE) | -0.964  (0.135) | 0.963  (0.134) | -0.038  (0.007) | 0.928  (0.324) |
| *Quadratic model* | | | | |
| R^2^ | 0.487 *** | 0.487 *** | 0.441 *** | 0.519 *** |
| β_0_ (SE) | 180.889  (11.3) | 85.526  (8.9) | 189.496  (11.5) | 325.502  (42.1) |
| β_1_ (SE) | -0.59  (0.57) | 1.324  (0.54) | -0.089  (0.021) | -9.313  (1.56) |
| β_2_ (SE) | -0.004  (0.005) | -0.004  (0.005) | 1.97E-5  (8E-6) | 0.088  (0.077) |

**Extended Data Fig. 1. *Parameters of linear and quadratic regression models for predicting thrombus relaxation times.*** **a** and **b**, coefficient of determination (R^2^) and regression coefficients (β) of linear and quadratic models for predicting the T1 (**a**) and T2 (**b**) relaxation times of clots based on the clot red blood cell (RBC), fibrin, iron, or water contents. Standard error (SE) of each regression coefficient value in brackets. P-values: * < 0.01; ** < 0.001; *** < 0.0001. Example of linear model prediction of relaxation time using RBC: Time (ms) = β1 * RBC% + β0; for the quadratic model: Time (ms) = β1 * RBC% + (β2 *RBC*RBC) + β0

**Extended Data Fig. 2.**

**Part 1: Primary and secondary biomarkers for T1 relaxation times.**

**
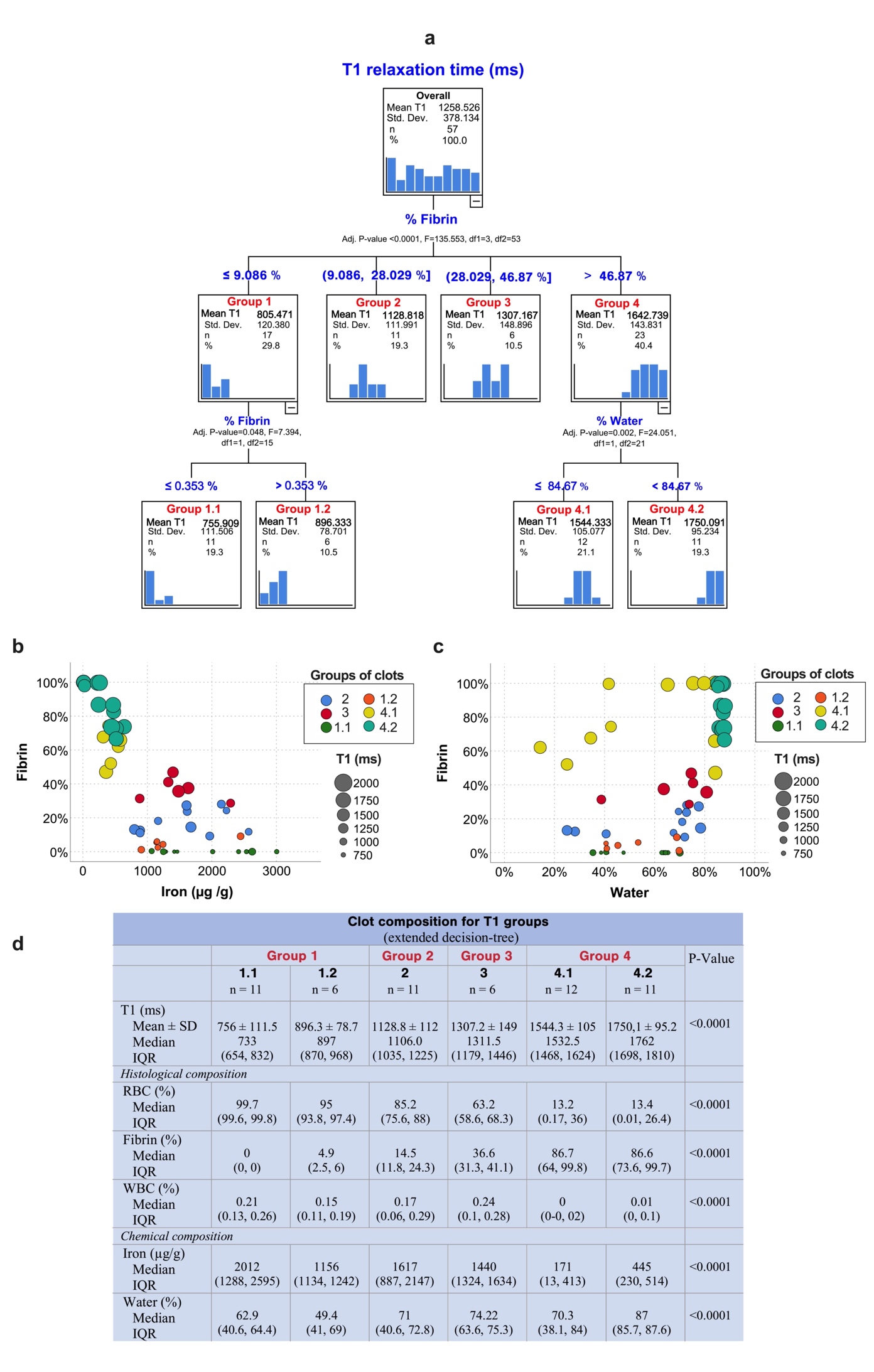
**

**Extended Data Fig. 2. Part 1**: ***Primary and secondary biomarkers for T1 relaxation times*.**

**a**, Extended decision tree allowing further subgroup formation at a significance level of less than 0.05. **b**, Bubble plot of T1 times concerning the fibrin and iron composition. **c**, Bubble plot of T1 times depending on fibrin and water contents. Groups or “nodes” created shown in different colors. Bubble size represents T1 times (ms). **d**, Table presenting the composition of each final “node”. Nodes that have been re-defined into further subgroups at a subsequent level no longer exist as an independent group (or “final node”) and therefore are not listed in this table. Results are expressed as medians and interquartile ranges (IQR). P-Values: Kruskal-Wallis Test.

**Extended Data Fig. 2.**

**Part 2**: **Primary and secondary biomarkers for T2 relaxation times.**


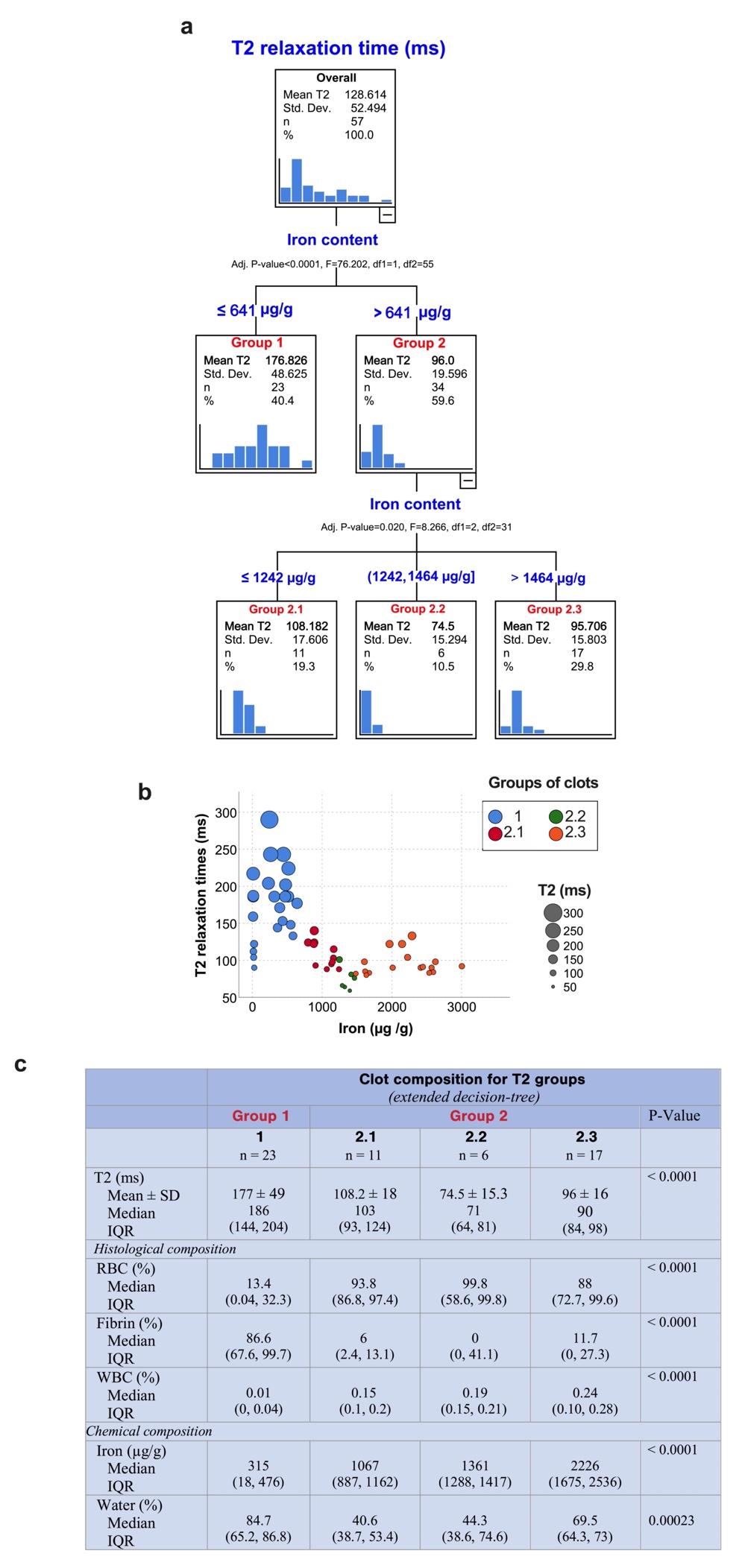


**Extended Data Fig. 2. Part 2**: ***Primary and secondary biomarkers for T2 relaxation times*.**

**a**, Extended decision tree for T2 times allowing further subgroup formation at P < 0.05. **b**, Bubble plot of fibrin and iron composition, with bubble size representing clot T2 relaxation time (ms). Different clot groups (or "nodes") shown in different colors. **c**, Insights into the clot composition of each "final node" of this decision tree. Nodes that have been refined into further subgroups at a subsequent level no longer exist as an independent group and therefore are not listed in this table. P-Values: Kruskal-Wallis Test.

**Extended Data Fig. 3.**


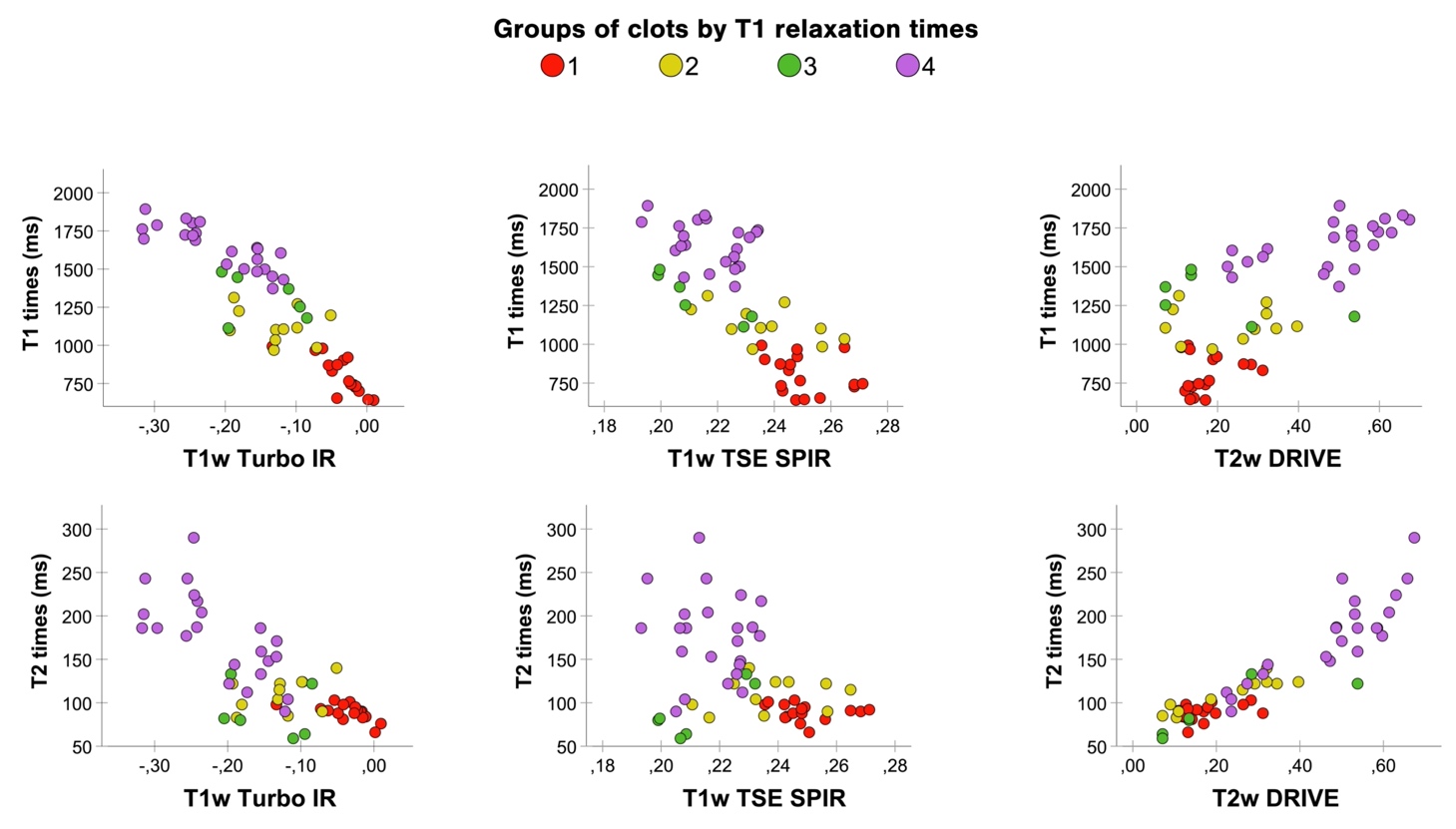


**Extended Data Fig. 3. *T1 and T2 relaxation times for clot* *signal intensity by sequence.*** Dot plots are grouped by clot classification T1 times (single-level depth classification from Fig. 2 in the main manuscript) and shown in color: group 1 (in red, n = 17); group 2 (in yellow, n = 11); group 3 (in green, n = 6); and group 4 (in violet, n = 23) with fibrin content cut-offs of ≤ 9.1 %, 9.1 – ≤ 28 %, 28 – 47 %, and >47 %, respectively. **a,** Distribution of clot signal intensity (x-axis) in T1w Turbo Inversion Recovery (T1w Turbo IR) with respect to the T1 relaxation time. **b,** Intensity values of clots in T1-weighted turbo spin echo with fat suppression using Spectral Presaturation inversion recovery (T1w TSE SPIR) with T1 relaxation times. **c,** Clot signal intensity in T2-weighted turbo spin echo with DRIven Equilibrium magnetization refocusing (T2w DRIVE) with T1 times. **d, e, and f,** similarly, dot plots of clot signal intensities measured in T1w Turbo IR (d), T1w TSE SPIR (e), and T2w DRIVE with respect to the T2 relaxation time values (f).
